# Kallistatin Attenuates Experimental Autoimmune Uveitis by Inhibiting Activation of T Cells

**DOI:** 10.3389/fimmu.2020.00975

**Published:** 2020-05-21

**Authors:** Fauziyya Muhammad, Priscilla N. Avalos, M. H. Mursalin, Jian-Xing Ma, Michelle C. Callegan, Darren J. Lee

**Affiliations:** ^1^Department of Microbiology and Immunology, University of Oklahoma Health Sciences Center, Oklahoma City, OK, United States; ^2^Department of Ophthalmology/Dean McGee Eye Institute, University of Oklahoma Health Sciences Center, Oklahoma City, OK, United States; ^3^Department of Physiology, University of Oklahoma Health Sciences Center, Oklahoma City, OK, United States; ^4^Oklahoma Center for Neuroscience, University of Oklahoma Health Sciences Center, Oklahoma City, OK, United States

**Keywords:** autoimmune disease, autoimmune uveitis, kallistatin, experimental autoimmune uveitis, T cells

## Abstract

Experimental autoimmune uveoretinitis (EAU) is a mouse model of human autoimmune uveitis. EAU spontaneously resolves and is marked by ocular autoantigen-specific regulatory immunity in the spleen. Kallikrein binding protein (KBP) or kallistatin is a serine proteinase inhibitor that inhibits angiogenesis and inflammation, but its role in autoimmune uveitis has not been explored. We report that T cells activation is inhibited and EAU is attenuated in human KBP (HKBP) mice with no significant difference in the Treg population that we previously identified both before and after recovery from EAU. Moreover, following EAU immunization HKBP mice have potent ocular autoantigen specific regulatory immunity that is functionally suppressive.

## Introduction

Uveitis is the third leading cause of blindness in Western countries, with an incidence between 25.6–122 cases per 100,000 a year, and a prevalence of 69–623 cases per 100,000 (1–4). While anterior uveitis is the most common form of uveitis, with 33% of these patients becoming chronic (5), posterior uveitis as the second most common form of uveitis is far more devastating. Approximately 17.6% of active uveitis patients experience transient or permanent vision loss (6), so a proper diagnosis and treatment are crucial for maintaining vision. Corticosteroids are the first line of treatment for uveitis patients, but due to the myriad of side-effects they are not a long-term treatment option (7–9). Therefore, immunosuppressive medications are used to control uveitis, with the goal of sustained remission (10–15). However, not all the immunosuppressive medications are effective and some chronic uveitis patients fail multiple treatment regimens. As such, additional immunosuppressive treatment options are necessary for the treatment of autoimmune uveitis.

Kallistatin (or kallikrein-binding protein) is a serine proteinase inhibitor that is encoded by *SERPINA4* (16, 17). Kallistatin was first identified as an inhibitor of tissue kallikrein (16, 18). Later, kallistatin has been investigated extensively for its role in diabetes (17, 19, 20). Type 1 diabetic patients have high serum levels of kallistatin (19), but the vitreous of diabetic retinopathy patients have low levels of kallistatin (19, 21). It has also been demonstrated that kallistatin has potent anti-inflammatory properties (22, 23). Therefore, it is of interest to determine if kallistatin could be a therapeutic alternative for autoimmune uveitis.

The most widely used model of human posterior autoimmune uveitis is the mouse model, experimental autoimmune uveitis (EAU). In the B10.RIII background, the disease is more severe and resolves more quickly compared to the more chronic C57BL/6J model. The onset of EAU is 12–14 days after immunization and resolution spontaneously occurs without relapse at 2–3 months after immunization (24–26). At resolution of EAU (post-EAU), regulatory immunity emerges in the spleen (27) and provides resistance to EAU during re-immunization and when adoptively transferred to mice that are immunized for EAU (27–30). Post-EAU regulatory immunity requires a regulatory T cell (Treg) that has been identified as CD4^+^CD25^+^Nrp-1^−^PD-1^+^PD-L1^+^Foxp3^+^ (29, 30).

A transgenic mouse that expresses human kallistatin under a chicken ß-actin promoter (TG-HKBP) has been studied with respect to angiogenesis related to diabetes (31). As such, in this report we asked if kallistatin over-expression affects T cell activation, provides resistance to EAU, and if regulatory immunity that provides resistance to EAU emerges in the spleen of post-EAU TG-HKBP mice. Our observations demonstrate that kallistatin over-expression provides resistance to EAU and does not affect the induction of regulatory immunity in the spleen of post-EAU TG-HKBP mice.

## Materials and Methods

### Mice

All mouse procedures described in this study were approved by the University of Oklahoma Health Sciences Center Institutional Animal Care and Use Committee (OUHSC IACUC) and all mouse study methods were carried out in accordance with the relevant guidelines approved by the OUHSC IACUC. C57BL/6J mice were purchased from Jackson Laboratories. Transgenic mice expressing human kallikrein binding protein under the chicken β-actin promoter (TG-HKBP or HKBP) were generated in the Jian-Xing Ma Lab on a C57BL/6J background (31, 32). All TG-HKBP mice were genotyped to verify transmission of the transgene before inclusion in this study. Serum was previously collected from TG-HKBP mice to verify elevated expression (31). All mice were housed in the Dean McGee Eye Institute vivarium under specific-pathogen-free conditions in a 12-h light cycle with access to food and water *ad libitum*. When mice were purchased from Jackson Laboratories they were allowed 2 weeks to acclimate under conventional housing conditions before inclusion in experimental procedures. The methods of euthanasia of all mice were approved by OUHSC IACUC and are consistent with the IACUC policies and the 2013 AVMA Guidelines on Euthanasia. Euthanasia of all mice for this study was done with carbon dioxide or cervical dislocation followed by a secondary method such as decapitation, bilateral thoracotomy, exsanguination, or vital organ removal. Mice were randomly chosen for experimental groups.

### Experimental Autoimmune Uveoretinitis (EAU)

EAU was induced in 6 to 10-week-old mice using the previously described immunization procedure (30). Complete Freund's adjuvant (CFA) with 5 mg/mL desiccated *M. tuberculosis* (Difco Laboratories, Detroit, MI) was emulsified with 2 mg/ml interphotoreceptor retinoid binding protein (peptides 1–20) (IRBP) (Genscript, Piscataway, NJ) to immunize mice for EAU. In order to minimize suffering mice were anesthetized with a ketamine/xylazine (100 mg/10 mg per kg) before immunization. The emulsion was injected subcutaneously at a volume of 200 μL total into two sites in the lower back followed by an intraperitoneal injection of 0.3 μg pertussis toxin. The course of EAU was evaluated every 3–4 days by fundus examination with a slit lamp microscope. Prior to examination of the retina, the iris was dilated with 1% tropicamide, and the cornea was flattened with a glass coverslip. We utilize a clinical scoring system that is validated by at least two members of the lab rather than histological examination because we have an interest in the resolution phase of EAU, so collecting mice over the course of disease for histological evaluation would significantly reduce the power of the study. As previously described, the clinical signs of observable infiltration and vasculitis in the retina were scored on a 5-point scale (33). Because the immunization procedure can elicit a score of 1, when the EAU score is 1 or lower, the disease is considered background or resolved. Both eyes were scored and the higher score was used to represent that mouse for that day, because the more severe score suggests that the systemic immune response is severe enough to elicit the greater score. The average score for the group of mice was then calculated. The highest score for each mouse over the entire course of disease was also determined and plotted for each group. Another masked member of the lab with experience evaluating EAU confirmed the clinical scores.

### *In vitro* Stimulation

A single cell suspension that was depleted of red blood cells using RBC lysis buffer (Sigma, St Louis, MO) was made from the spleens collected into 5% FBS in RPMI supplemented with 10 μg/ml Gentamycin (Sigma), 10 mM HEPES (GE Healthcare), 1 mM Sodium Pyruvate (BioWhittaker), Nonessential Amino Acids 0.2% (BioWhittaker). Serum free media (SFM) consisting of RPMI-1640 with 1% ITS+1 solution (Sigma) and 0.1% BSA (Sigma) was used to resuspend the spleen cells with IRBP (50 μg/mL) or α-CD3 (clone 2C11, Biolegend). The spleen cells were then incubated for 48 h at 37°C and 5% CO_2_ to reactivate antigen specific T cells. After the reactivation cells were collected for flow cytometry analysis or adoptive transfer (1 × 10^6^) into recipient mice.

### Intraocular Injection

Mice were anesthetized using isoflurane. CD4^+^ splenocytes (1 × 10^6^/μL) were injected into the left and right eye of each mouse with a sterile borosilicate glass micropipette (Kimble Glass Inc., Vineland, NJ, USA) beveled to an approximate bore size of 10–20 μm (BV-10 KT Brown Type micropipette beveller, Sutter Instrument Co., Novato, CA, USA). The micropipettes were inserted just posterior to the superior limbus under stereomicroscopic visualization, and 1-μL volume was injected directly into the vitreous. Injection rates and volumes were monitored using a programmable cell microinjector (Microdata Instruments, Plainfield, NJ, USA).

### Flow Cytometry

Mouse spleen cells were washed with PBS with 1% BSA (staining buffer), blocked with mouse IgG in staining buffer, then stained with conjugated antibodies. Antibodies used were anti-CD4 (clone RM4-5, Biolegend, San Diego, CA), anti-CD25 (clone PC61, Biolegend), anti-PD-1 (clone 29F.1A12, Biolegend), anti-PD-L1 (clone 10F.9G2 Biolegend), anti-Nrp-1 (Cat FAB5994N, R&D Systems, Minneapolis, MN), anti-FoxP3 (clone FJK-16s, eBiosciences), anti-CD44 (clone IM7, Biolegend), anti-CD62L (clone MEL-14, Biolegend), anti-CD45.1 (clone A20, Biolegend), and anti-CD45.2 (clone 104, Biolegend).

Stained cells were analyzed in the Oklahoma Medical Research Facility (OMRF) Flow Cytometry Core Facility on a BD LSRII (BD Biosciences) data was analyzed using FlowJo Software (Tree Star, Inc., Ashland, OR).

### Statistics

Statistical significance between maximum EAU scores and flow cytometry results was determined using non-parametric Mann-Whitney two-tailed test. Two-way ANOVA was also used to assess significant changes in the tempo of disease between the groups of treated EAU mice with post-test Bonferroni comparison analysis. Statistical significance was designated when *P* ≤ 0.05 and analyzed using Prism 6 (GraphPad Software, Inc., La Jolla, CA).

## Results

### Kallistatin Attenuates EAU

**Figure 1:**
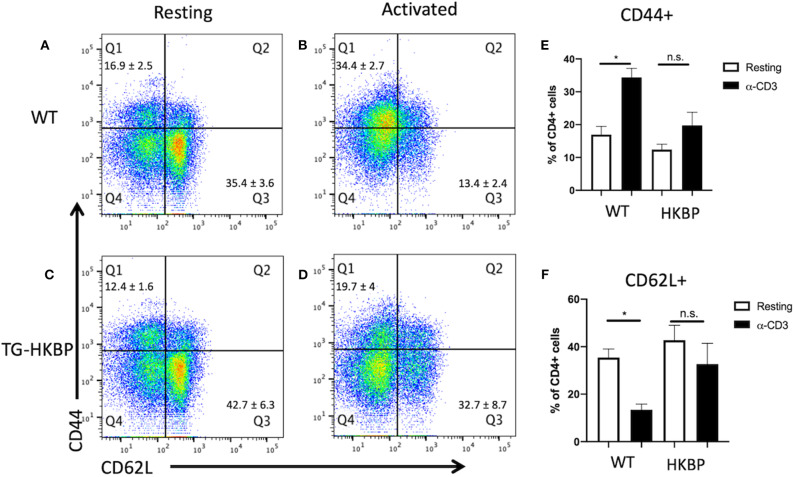
T cell activation in T cells from TG-HKBP mice. Wild-type and TG-HKBP splenocytes were cultured ± α-CD3 for 2 days, stained for CD4, CD44, and CD62L, and analyzed by flow cytometry. Representative dot plots are shown for wild-type (*n* = 5) **(A,B)** and TG-HKBP (*n* = 7) **(C,D**), and bar graphs show the mean ± SEM for the indicated group **(E,F)**. One to three mice were used for each experiment, and each experiment was repeated three times. Statistical significance (*P* ≤ 0.05) is designated by *, n.s. indicates no significance.

**Figure 2:**
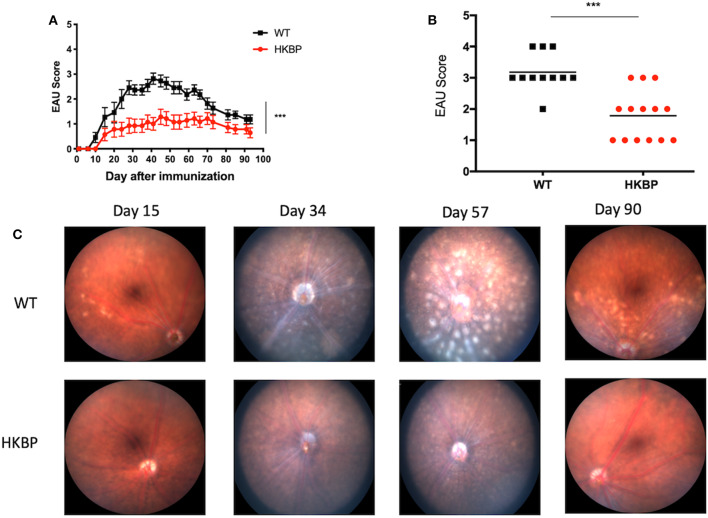
EAU course of disease and maximum scores in TG-HKBP mice immunized for EAU. Wild-type and TG-HKBP mice were immunized for EAU and the clinical EAU score was assessed by slit lamp examination of the retina every 3–4 days. The highest score for each mouse on the indicated day was averaged with all the mice on that day and graphed with the SEM for WT (black squares, *n* = 11) and HKBP mice (red circle, *n* = 14) **(A)**. The highest EAU score for each mouse over the course of EAU was also determined (black squares, *n* = 11) and HKBP mice (red circle, *n* = 14) **(B)**. Representative images at the indicated time points are shown **(C)**. Statistical significance (*P* ≤ 0.05) is designated by ***.

We first asked if there is a defect in the T cell response in TG-HKBP mice. Naïve and memory T cells were evaluated in splenic T cells that were non-specifically activated through CD3. As expected, wild-type T cells showed a significant elevation in memory T cells (CD44+) and significant reduction in naïve T cells (CD62L+) (Figures 1A,B,E,F). In contrast TG-HKBP T cells showed no significant change in memory or naïve T cell populations following CD3 activation (Figures 1C–F). This inhibition of T cell activation and the anti-inflammatory properties of kallistatin (kallikrein-binding protein) (23) prompted us to ask if it suppresses experimental autoimmune uveitis (EAU). TG-HKBP mice have been previously characterized in studies related to diabetic retinopathy, and serum kallistatin in these mice is over four-fold higher than the endogenous mouse kallistatin (31, 32). When TG-HKBP mice were immunized for EAU, a significant decrease in the severity and acceleration of resolution was observed in TG-HKBP mice compared to wild-type mice immunized for EAU (Figure 2A). Because the tempo of disease can vary from mouse to mouse over the course of EAU, we also compared the maximum severity of each mouse over the entire course of disease by plotting the maximum score of each mouse (Figure 2B). The maximum EAU score also showed that TG-HKBP mice experienced significantly less severe EAU compared to wild-type mice (Figure 2B). Because the immunization procedure can elicit a score of 1, when the EAU score is 1 or lower, the disease is considered resolved. These observations demonstrate that kallistatin attenuates EAU and accelerates the resolution of EAU. Our clinical scores were also confirmed with fundus photos (Figure 2C). These observations demonstrate that kallistatin inhibits T cell activation resulting in the attenuation of EAU.

### Kallistatin Does Not Significantly Change the Treg Compartment

**Figure 3:**
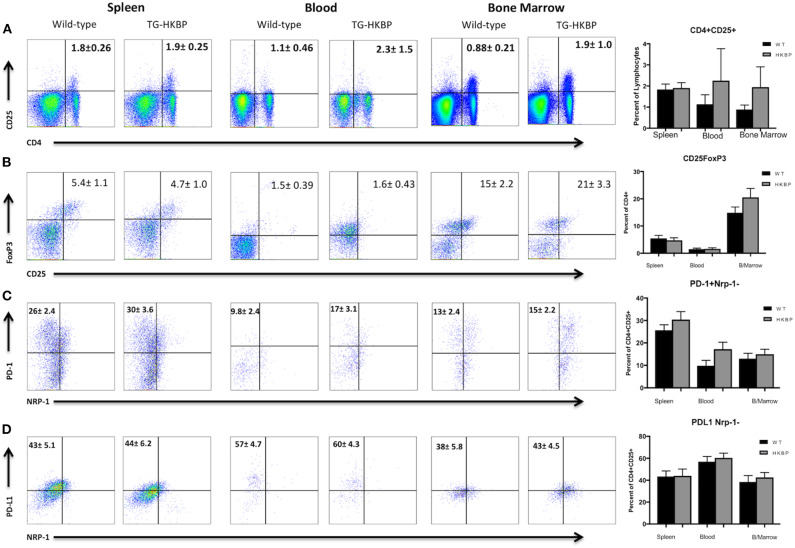
Flow cytometry analysis of TG-HKBP mice for Treg cells. Spleen, blood, and bone marrow T cells from WT and TG-HKBP mice were stained for Treg cell markers. Representative flow plots with the mean and SEM of 3 independent experiments of 3–4 mice per experiment of WT mice (*n* = 10, black bars) and HKBP mice (*n* = 11, gray bars). CD4 and CD25 staining on T cells gated on the lymphocyte population based on FSC and SSC **(A)**, CD25 and FoxP3 staining of cells gated on CD4 **(B)**, NRP-1 and PD-1 staining of cells gated on CD4 and CD25 **(C)**, NRP-1 and PD-L1 staining of cells gated on CD4 and CD25 **(D)**.

We next sought to ask if the TG-HKBP mice have alterations in the Treg compartment. TG-HKBP mice have significantly less circulating B cells and a significantly greater percentage of circulating CD4^+^ T cells (32). Regulatory T cells (Tregs) are necessary for the resolution of EAU (34) and for providing resistance to relapse (30, 35). Because we observed attenuated EAU in TG-HKBP mice, we asked if there is a change in the Treg compartment. Because the Tregs that provide resistance to relapse have been identified as CD4^+^CD25^+^Nrp-1^−^PD-1^+^PD-L1^+^Foxp3^+^ (30), we asked if TG-HKBP mice had an alteration in the number of these Tregs. The blood and bone marrow showed a trend toward an increase in PD-1^+^PD-L1^+^Foxp3^+^Nrp-1^−^CD25^+^CD4^+^ Tregs, but not a statistically significant increase (Figures 3A–D). These observations suggest suppression of EAU by TG-HKBP mice may be due to suppression of an inflammatory response but not necessarily an increase in the induction of Tregs.

### Post-EAU Treg Cells Emerge in the Spleen of HKBP Mice

**Figure 4:**
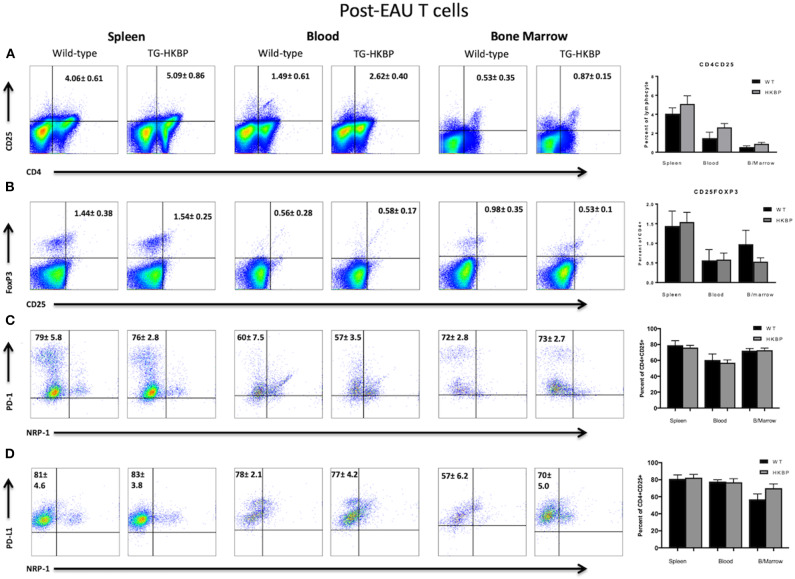
Flow cytometry analysis of post-EAU TG-HKBP mice for Treg cells. Spleen, blood, and bone marrow T cells from WT and TG-HKBP mice were stained for Treg cell markers. Representative flow plots with the mean and SEM of 3 independent experiments of 3–7 mice per experiment of WT mice (*n* = 6, black bars) and HKBP mice (*n* = 15, gray bars). CD4 and CD25 staining on T cells gated on the lymphocyte population based on FSC and SSC **(A)**, CD25 and FoxP3 staining of cells gated on CD4 **(B)**, NRP-1 and PD-1 staining of cells gated on CD4 and CD25 **(C)**, NRP-1 and PD-L1 staining of cells gated on CD4 and CD25 **(D)**.

The post-EAU regulatory immunity that emerges in the spleen of mice at resolution of EAU provides resistance to relapse (29, 30, 34, 35), so that may be a mechanism to provide sustained remission for uveitis patients in the clinical setting. A requirement of post-EAU regulatory immunity is resolution of EAU (27). Because the clinical EAU scores of TG-HKBP mice were significantly lower compared to wild-type mice, we therefore asked if the post-EAU TG-HKBP mice also had CD4^+^CD25^+^Nrp-1^−^PD-1^+^PD-L1^+^Foxp3^+^ Tregs in the spleen at resolution of EAU. The spleens from post-EAU TG-HKBP mice were collected and reactivated *in vitro* as has been done before (29, 30, 35). There was no significant difference between post-EAU wild-type and post-EAU TG-HKBP Treg cell populations in the spleen (Figures 4A–D). However, the percentage of PD-1 and PD-L1 expressing CD4^+^CD25^+^ T cells (Figures 4C,D) was increased compared to the unimmunized unstimulated T cells (Figures 3C,D). Because there is no significant difference between the post-EAU wild-type mice and TG-HKBP mice, these observations demonstrate that this post-EAU Treg population is present in the spleen of TG-HKBP mice.

### Kallistatin Does Not Affect the Emergence of Post-EAU Regulatory Immunity

**Figure 5:**
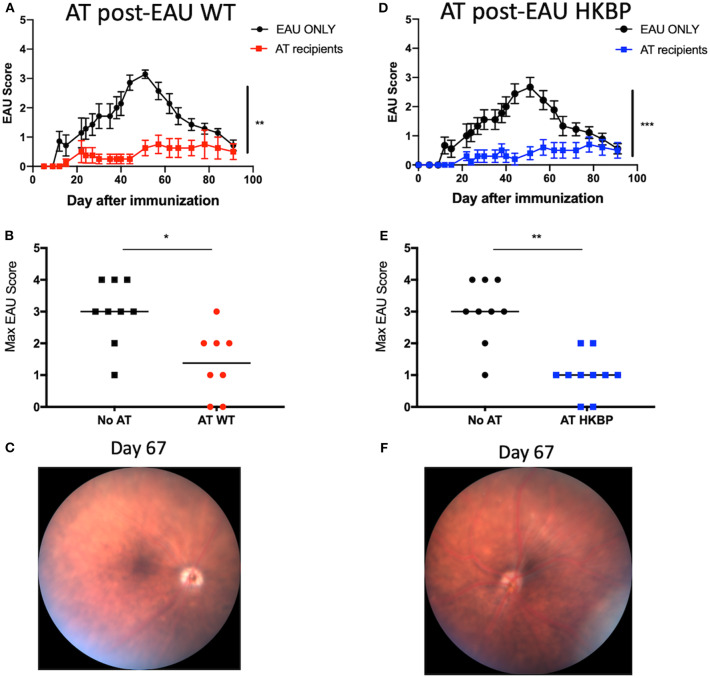
EAU course of disease in recipient mice immunized for EAU that received splenocytes from post-EAU TG-HKBP mice. Post-EAU splenocytes from WT or TG-HKBP mice were reactivated *in vitro* and transferred to recipient mice that were immunized for EAU. Shown are clinical scores and representative retina images from recipient mice. Recipient scores are compared with EAU mice that did not receive cell transfer (black circles and squares). Mice that received WT post-EAU splenocytes (red squares and circles, *n* = 8) **(A,B)** and mice that received HKBP post-EAU splenocytes (blue squares *n* = 10) **(D,E)**. The same group of control EAU mice that did not receive a transfer are used for comparison **(A,B,D,E)**. A representative fundus image is shown for mice that received an adoptive transfer **(C,F)**. Statistical significance is designated by * (*P* ≤ 0.05), ** (*P* ≤ 0.01), and *** (*P* ≤ 0.005).

We next asked if the post-EAU regulatory immunity in TG-HKBP mice is functionally suppressive. The spleen of post-EAU TG-HKBP mice was collected and reactivated *in vitro* as has been done before (29, 30, 35). The reactivated splenocytes were then adoptively transferred to recipient mice immunized for EAU. As expected, mice that received splenocytes from post-EAU wild-type mice showed a significant suppression of EAU (29, 30, 33) compared to EAU mice that did not receive an adoptive transfer (Figures 5A,B). The mice that received post-EAU TG-HKPB splenocytes also showed a significant suppression of EAU (Figure 5D) and significantly lower maximum EAU scores compared to controls (Figure 5E). A representative fundus photo is shown at day 67 to confirm the clinical observations (Figures 5C,F). These observations demonstrate that kallistatin does not affect the emergence of post-EAU regulatory immunity in the spleen.

### Kallistatin Promotes Tregs in an Intrinsic Manner

**Figure 6:**
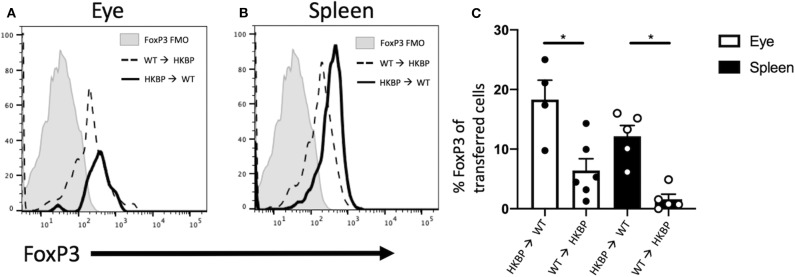
FoxP3 expression in transferred HKBP or WT cells transferred to WT or HKBP mice. CD4^+^ splenocytes were collected from donor HKBP or WT mice and transferred to recipient congenic mice. Donor cells were injected into the eyes (1 × 10^5^ cells) or intravenously (1 × 10^6^ cells). Forty eight-hours after the transfer the eyes were collected from mice that received an intraocular injection or the spleen was collected from mice that received an intravenous injection. Cells were then analyzed by flow cytometry for CD45.1, CD45.2, and FoxP3. Histograms shown are representative for all experiments, FoxP3 expression in the transferred cells collected from injected eyes **(A)**, or FoxP3 expression in the transferred cells collected from the spleen of intravenously injected mice **(B)**, the same fluorescence minus one (FMO) control is shown in both panels and is from the spleen. The mean ± SEM for all experiments are shown **(C)**. Each experiment included 1–3 mice and the experiment was repeated three times. Statistical significance (*P* ≤ 0.05) is designated by *.

In order to determine if kallistatin mediated Treg induction is intrinsic or extrinsic, we transferred CD4^+^ HKBP cells into wild-type mice to determine if FoxP3 expression is mediated through intrinsic T cell expression of kallistatin. Recipient mice were congenic (CD45.1) to identify the CD45.2 HKBP transferred cells. We found a significant elevation of FoxP3 expression in the transferred cells when HKBP cells were transferred to wild-type mice compared to wild-type cells transferred to the eyes of HKBP mice (Figures 6A,C). These observations show that kallistatin functions intrinsically in T cells. We next asked if this was an ocular specific effect by transferring congenic cells intravenously and assaying the spleen for FoxP3 expression in the transferred T cells. A similar pattern of expression with ocular injected cells was observed with a significant increase in FoxP3 expression in HKBP cells transferred to wild-type mice compared with wild-type cells transferred to HKBP mice (Figures 6B,C). These observations demonstrate that kallistatin functions intrinsically on T cells to maintain or induce Treg cells.

## Discussion

It has been previously demonstrated that a relationship between kallistatin and inflammation exists. This further illustrates a relationship between angiogenesis and inflammation. Previous reports demonstrate that TG-HKBP mice exhibit a reduction in B cells and a greater percentage of CD4^+^ T cells (32). It has also been previously shown that TG-HKBP mice also have an impairment of wound healing due to defects in angiogenesis (17). In this report, we asked if kallistatin is capable of suppressing autoimmune uveitis. Our observations demonstrate that EAU is attenuated in TG-HKBP mice and display a reduction in severity and acceleration of resolution. This indicates the anti-inflammatory and anti-angiogenic properties of kallistatin are sufficient to suppress autoimmune uveitis and should be further investigated to develop novel treatments for autoimmune uveitis. However, the anti-angiogenic properties associated with elevated systemic kallistatin will need to be carefully considered if this is translated into a uveitis treatment. Another caveat to consider is that the overexpression of kallistatin is not equivalent to administration of the protein systemically because kallistatin likely has different properties in the eye compared to systemic kallistatin (17, 19, 21). Therefore, it may be the case that kallistatin given locally into the eye may be a suitable alternative to systemic administration.

It has been demonstrated that kallistatin is a Wnt/β-catenin inhibitor. This accounts for the reduction of lymphocytes because of the reduction of hematopoietic stem cells (32, 36–38). A reduction in T cells supports the hypothesis that EAU is suppressed because of a lack of effector T cells that can mediate inflammation. This may still be the case, but because we observe Treg cells that emerge at the resolution of EAU in TG-HKBP mice, it is likely that there are still pathogenic effector T cells being generated. However, given the anti-inflammatory properties and antagonism of TNF-α (23, 39) kallistatin has the capacity to limit the extent to which these effector T cells can mediate disease. Support for a kallistatin-mediated limitation of T cell activation is provided with the significant reduction of CD44+ TG-HKBP T cells following CD3 activation. As such, further studies are necessary to determine the exact mechanism whereby, kallistatin suppresses EAU.

Since mice that recover from EAU have regulatory immunity that provides resistance to EAU, it was of interest to determine if the post-EAU TG-HKBP mice also generate this regulatory immunity (29, 30, 35). Our observations demonstrate that not only is EAU attenuated in TG-HKBP mice, but they also generate regulatory immunity that is capable of providing resistance to relapse. The importance of this discovery is that if a uveitis patient is treated with kallistatin, there is still a generation of regulatory immunity that could provide sustained remission. However, overexpression of kallistatin is not equivalent to the administration of the protein as a therapeutic, but it has been demonstrated to confer greater survival in a sepsis model (40). Therefore, additional study assessing the efficacy of recombinant kallistatin is needed. We further show that the Tregs that emerge in the spleen of TG-HKBP mice share similar markers as the typical Tregs that normally emerge during EAU, so the Tregs likely emerge through similar mechanisms.

Because the TG-HKBP mice express serum levels similar to diabetic patients with vascular complications (17, 19), it may be the case that diabetic patients could be resistant to the development of autoimmune uveitis. However, because there are many mechanisms that promote autoimmune uveitis, this may only represent one such mechanism that provides resistance. As such, it has been observed that patients with diabetic retinopathy experience exacerbated uveitis (41, 42). This discrepancy is likely due to the status of ocular immune privilege, as is observed in diabetic retinopathy patients with lower kallistatin concentration in the vitreous (19, 21), and if systemic regulatory immunity has been established or not. In the case that ocular immune privilege has established regulatory immunity to protect against ocular inflammation, the patient would be protected against uveitis.

These observations demonstrate for the first time to our knowledge that kallistatin is effective in decreasing the severity of EAU and in accelerating the resolution of EAU. This suppression is likely due to multiple mechanisms that may include an inhibition of T cell activation and an intrinsic predisposition to regulatory activity in T cells. Importantly, in mice that overexpress kallistatin and have recovered from EAU, a functionally suppressive regulatory immunity is found in the spleen. This regulatory immunity in the spleen has a similar abundance of post-EAU Tregs that naturally emerge at resolution of EAU. Therefore, kallistatin suppresses autoimmune uveitis while also allowing for the emergence of systemic regulatory immunity that provides resistance to EAU.

## Data Availability Statement

The datasets generated for this study are available on request to the corresponding author.

## Ethics Statement

This animal study was reviewed and approved by Institutional Animal Care and Use Committee, University of Oklahoma Health Science Center.

## Author Contributions

All experiments, analysis, and experimental design of this work were done by DL, PA, FM, MM, MC, and J-XM. The conceptual design of this work and the writing of this manuscript was a collaborative effort between DL and J-XM.

## Conflict of Interest

The authors declare that the research was conducted in the absence of any commercial or financial relationships that could be construed as a potential conflict of interest.
